# Supplementary material for: Indirect and suboptimal control of gene expression is widespread in bacteria
Source: Mol Syst Biol. 2013 Apr 16;9:660. doi: 10.1038/msb.2013.16 (PMC3658271; doi:10.1038/msb.2013.16)
Supplement: Supplementary Information — Supplementary Figures S1–2 [file msb201316-s1.pdf]

Supplementary information for
"Indirect and Suboptimal Control of Gene Expression is Widespread in Bacteria"

Table of Contents

- Supplementary Figure 1: Optimal control under constant or unpredictably-varying conditions.
- Supplementary Figure 2: Matched measurements of mutant fitness and gene expression in Shewanella oneidensis MR-1.
- Dataset 1: Fitness data for Shewanella oneidensis MR-1.
- Dataset 2: Pairs of functionally-related genes in Shewanella oneidensis MR-1 that are not in the same operon and are not coexpressed.

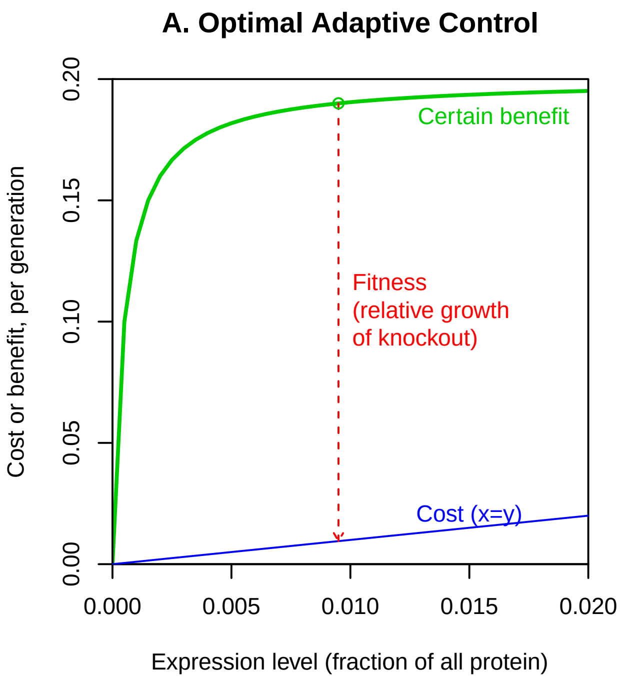

Supplementary Figure 1 – Optimal control under constant or unpredictably-varying conditions. We consider a protein with a maximum benefit (not including the cost of expression) of 0.2 per generation and which reaches half-maximal benefit at 0.0005 of protein (solid green line). The parameters are arbitrary, but we assume that the benefit of the protein saturates because this arises naturally in metabolic models and has been confirmed experimentally (D. Fell, "Understanding the Control of Metabolism," Portland Press, 1997). (Intuitively, once a metabolic enzyme is highly expressed, increasing its expression further will increase the concentration of its product, but unless the expression of downstream enzymes is increased as well, downstream enzymes will become saturated and total flux through the pathway will only increase slightly.) We conservatively assume that the protein has no detrimental activity when unneeded and that its cost of protein production is equal to the amount of protein (blue line). (A) Optimal adaptive control when conditions rarely change. The optimum expression maximizes benefit minus cost (green circle), and the fitness cost of disabling the protein is the difference between the two (red arrow). But when the protein is not beneficial, the optimal expression level is 0. (B) Optimal "standby" control under unpredictably varying conditions. We consider two situations with unpredictable future changes in conditions. In the first situation, the protein is currently beneficial, but this may not continue, so on average, the benefit is just 80% of the benefit under constant conditions (dashed green line). In the second situation, the protein is currently not beneficial, but conditions may change, so on average, the benefit is 20% of the benefit under constant beneficial conditions (dotted green line). The expression level that maximizes benefit minus cost in each situation is shown (green circles). The range of expression is less than in (A), but the expression level still changes (black arrow). Intuitively, because the cost of expressing a small amount of unneeded protein is small relative to the potential benefit, it is beneficial to gamble on making a small amount of the protein. And because of diminishing returns to making more of the protein, the optimal expression level is higher when the benefit is higher. However, under constant conditions with no benefit, standby expression of the protein incurs a cost and fitness is reduced (red line).

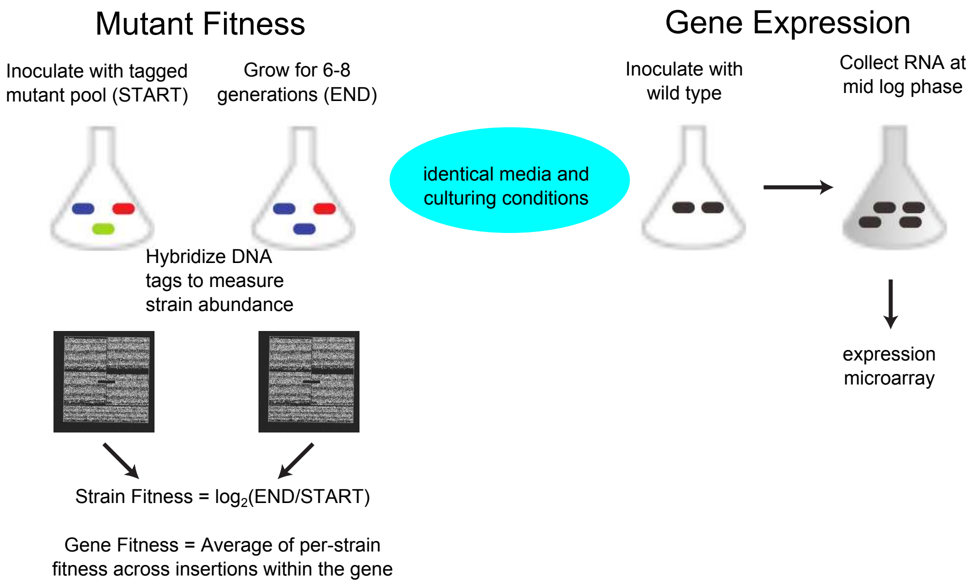

Supplementary Figure 2 – Matched measurements of mutant fitness and gene expression in Shewanella oneidensis MR-1. Each pool of mutants contains about 4,000 strains, and each strain has a transposon inserted at a different location in the genome and a tag that allows that strain to be distinguished from the other strains in that pool (Oh et al. 2010).

Dataset 1 – Fitness data for Shewanella oneidensis MR-1

The first tab shows the metadata for the fitness experiments; the Experiment number is the heading in the other tables. The remaining tabs show the per-strain fitness values, the per-gene fitness values, and the per-gene z scores. In these tables, the locusId is the MicrobesOnline ("VIMSS") identifier and the sysName is the LocusTag.

Dataset 2 – Pairs of functionally-related genes in Shewanella oneidensis MR-1 that are not in the same operon and are not coexpressed

We list pairs of genes that are cofit and in the same functional category (TIGR subrole) but are not in the same operon, near each other in the genome, or coexpressed. For each pair, we manually examined their annotations and their fitness patterns to determine if they truly had closely-related functions or not. For pairs of flagellar genes, we also report whether they are coregulated and in the same "class" in *Pseudomonas aeruginosa* according to Dasgupta *et al.* 2003. The data is provided as a tab-delimited file.
